# Who’s in Charge? Leadership during Epidemics, Bioterror Attacks, and Other Public Health Crises

**DOI:** 10.3201/eid1606.100345

**Published:** 2010-06

**Authors:** Patrick J. McConnon

**Affiliations:** Council of State and Territorial Epidemiologists, Atlanta, Georgia, USA

**Keywords:** epidemics, leadership, public health, bioterrorism, book review

Dr Laura Kahn has produced a useful book that provides a brief historical background on public health and terrorism, followed by interesting examples of leadership during outbreaks and events that escalated to public health crises. The roles of astute clinicians, public health professionals, appointed public health leaders, and elected officials are described by the players themselves. These insights provide important perspectives and are fascinating reading, but each event includes the voices of only a few of many participants. This omission may leave the reader hungry for a wider variety of viewpoints.

Kahn takes the reader through a thought-provoking overview of the complexity of leadership and some early milestones in public health. Kahn makes it clear that politics, economics, communications, and interpersonal relations are as central to today’s public health crises as they were in the past.

Persuasive examples support Kahn’s main thesis that political leadership is critical during a public health crisis, whether the crisis results from natural causes or from bioterrorism. Kahn says, “Questions about leaders and leadership have intrigued scholars in both Western and Eastern civilizations for centuries. Plato, Confucius, and Machiavelli all speculated about leaders… and the qualities of leadership.” Kahn concludes that 1) informed, engaged, and prepared elected officials are essential to effective response; 2) because crisis response decisions inevitably will be made in the absence of perfect information, leaders require judgment and common sense; 3) elected and appointed leaders must be effective; and 4) dual leadership during a crisis can cause confusion.

The author provides a convincing case for her conclusions with lively examples and first-hand accounts and offers several concrete suggestions to prepare elected officials for leadership roles. The same compelling case is not made for Kahn’s assertion of a “legal conundrum when dealing with the bioterrorism attack.” She suggests that the Centers for Disease Control and Prevention (CDC) should lead the public health response to such episodes but alleges that legal and organizational impediments hinder CDC from fulfilling that lead role.

Unquestionably, CDC must and does play a lead role during large-scale, multistate public health events. The legal and organizational impediments to fulfilling that role are not obvious to this reviewer, especially given CDC’s success in addressing many such crises. Kahn may be referring to impediments within the federal structure and chain of command. However, current law specifies the roles of CDC and the departments of Health and Human Services, and Homeland Security. CDC has ample legal authority to supplement its technical and scientific leadership during an emergency, especially when state and local capacities are outstripped.

Kahn suggests federalizing and centralizing the national response system through changes in the legal framework and organizational structures of the public health system, arguing that if CDC were organized for response as the Environmental Protection Agency or the Federal Bureau of Investigation is, delays, leadership confusion, and communication issues would be resolved. She identifies some leadership problems but fails to acknowledge the strong collegial relationship between state public health authorities and CDC that has produced innumerable successful responses to crises. Restructuring the traditional relationship between states and the federal government seems unnecessary.

This problem does not overshadow an otherwise informative and engrossing book. In an era of emerging infectious diseases, bioterrorism, and large-scale natural disasters, we will continue to have to address the types of events Dr. Kahn describes. Those involved in responding to such events would benefit from studying the lessons of the past to better manage future emergencies.

